# Characterization of HBV surface antigen isoforms in the natural history and treatment of HBV infection

**DOI:** 10.1097/HC9.0000000000000027

**Published:** 2023-04-04

**Authors:** Mary A. Rodgers, Pir A. Shah, Mark Anderson, Ana S. Vallari, Jeffrey Gersch, Dora Mbanya, Silvia Sauleda Oliveras, Saad Choudhry, Thomas P. Leary, Mary C. Kuhns, George J. Dawson, Gavin A. Cloherty, Daryl T.Y. Lau

**Affiliations:** 1 Infectious Diseases Research, Abbott Diagnostic, Abbott Park, Illinois, USA; 2 Liver Center, Beth Israel Deaconess Medical Center, Harvard Medical School, Boston, Massachusetts, USA; 3 University of Yaounde I, Haematology, Immunology, Microbiology and Infectious Diseases, Yaounde, Cameroon; 4 Banc de Sang I Teixits, Barcelona, Spain

## Abstract

The loss of HBV HBsAg or functional cure is a desirable goal of hepatitis B management. The relative abundances of HBsAg isoforms may offer additional diagnostic and predicting values. To evaluate the clinical utility of HBsAg isoforms, we developed novel prototype assays on the ARCHITECT automated serology platform that specifically detects total-HBsAg (T-HBsAg), large (L-HBsAg), and middle (M-HBsAg) products of the S gene to determine the isoform composition of human specimens from acute and chronic HBV infection and during long-term nucleos(t)ide analog therapy.

**Results:**

In the early phase of acute HBV infection, L-HBsAg and M-HBsAg emerged within days and were in parallel to T-HBsAg during the entire course of infection. M-HBsAg levels were consistently higher than L-HBsAg levels. Patients with HBeAg(+) chronic hepatitis B had higher T-HBsAg, M-HBsAg, and L-HBsAg levels compared with HBeAg(−) patients. Correlations of M-HBsAg and L-HBsAg to T-HBsAg were similar in both. In contrast, there was no strong correlation between L-HBsAg or M-HBsAg with HBV DNA levels. During long-term nucleos(t)ide analog treatment, changes in HBsAg isoform abundance were proportional to T-HBsAg regardless of treatment responses for both HBeAg(+) and HBeAg(−) chronic hepatitis B. A larger sample size may be necessary to detect a significant difference.

**Conclusion:**

HBsAg isoform compositions parallel T-HBsAg levels in both acute and chronic hepatitis B infection. L-HBsAg and M-HBsAg individual biomarkers do not appear to provide an additional diagnostic benefit for staging chronic disease or monitoring response to treatment with current therapies.

## INTRODUCTION

HBV HBsAg is the hallmark of HBV infection.^1^–^3^ HBsAg is expressed from a single open reading frame that encodes 3 isoforms of differing lengths; namely, the small (S-HBsAg), middle (M-HBsAg), and large (L-HBsAg).^4^ S-HBsAg is the predominant isoform in both infectious virions (Dane particles) and subviral particles (SVPs)^4^; it contains the epitopes that elicit protective immunity from natural infection and vaccination.^5^ In contrast to the S-HBsAg, the proportion of L-HBsAg and glycosylated M-HBsAg is much lower in SVPs than in virions.^4^ Limited reports on the composition of the HBsAg isoforms suggest that they may identify patients in the inactive phase of hepatitis B, are associated with liver cancer progression, or predict HBsAg loss.^6^–^10^

We applied the newly developed automated prototype HBsAg isoform serological tests to determine the composition of the L-HBsAg, M-HBsAg, and T-HBsAg (predominantly S-HBsAg) from clinical samples of 3 separate cohorts, namely, subjects with early acute infection, those in different phases of CHB, and patients on prolonged nucleos(t)ide analog (NA). An understanding of the relative concentrations of these HBsAg isoforms may provide clues to the eventual HBsAg clearance or functional cure.

## METHODS AND HUMAN SPECIMENS

### HBsAg isoform assays

Prototype HBsAg isoform assays were developed on the fully automated chemiluminescent immunoassay ARCHITECT instrument using the HBsAg NEXT assay format and diluents (Abbott Diagnostics).^11^ Antibodies directed against the “a” determinant loop shared by all isoforms were coated on the solid phase to capture HBsAg-containing particles. T-HBsAg, L-HBsAg, and M-HBsAg were detected with acridinium-conjugated isoform-specific antibodies.^12^ In parallel, an S-HBsAg detection antibody that was labeled at the same time as the L-HBsAg and M-HBsAg antibodies was used to detect T-HBsAg. Results were calculated as signal-to-noise (S/N) ratios using average relative light units detected using a pool of HBsAg-negative normal human plasma. A provisional cutoff was determined based on 10 SD of the S/N population mean of N=100 HBsAg-negative specimens for each isoform test, resulting in a specificity of 100%. Cutoff values were 2.0 S/N for L-HBsAg, 1.9 for M-HBsAg, and 2.1 S/N for T-HBsAg. For longitudinal antiviral treatment samples, results were also calculated as the percentage of baseline or T-HBsAg S/N. Since the isoform assays were qualitative, ratios could not be compared directly between L-HBsAg and M-HBsAg relative to each other. All specimens were determined to be HBsAg reactive on the ARCHITECT HBsAg Qual assay (List 4P53; Abbott Diagnostics).

HBsAg isoform dilutional linearity was tested using 2 commercially available HBsAg positive samples (053FW75918P and 019FG09747P) purchased from the American Red Cross. Briefly, each sample was initially diluted to 1:500 in normal human plasma to yield an expected quantitative HBsAg (qHBsAg) concentration that would fall within the linear range of the qHBsAg assay (<250 IU/mL). From this stock, 10 additional 1:2 serial dilutions were made in normal human plasma and each was tested for qHBsAg, total-S, M, and L isoforms. HBsAg isoform S/N results were compared with measured qHBsAg results.

### Other serological and molecular assays

HBV DNA was quantified by the m2000 RealTi*m*e HBV assay (List 2N40; Abbott Molecular Diagnostics) according to the package insert. HBV RNA testing was as described.^13^ HBsAg was measured by the quantitative ARCHITECT HBsAg assay (List 6C36; Abbott Diagnostics) according to the package insert. Specimens with >250 IU/mL were diluted in normal human plasma to a level below 250 IU/mL before testing with the quantitative HBsAg assay. Where necessary, qHBsAg results were corrected for the dilution factor before analysis. HBeAg data were generated with the ARCHITECT HBeAg assay (List 6C32; Abbott Diagnostics) according to the package insert.

### Clinical samples

#### Control materials

Recombinant subtype ay S-HBsAg was purified from HEK293 cell culture supernatants as described^11^ and diluted in HBsAg-negative normal human plasma. Dane particles were purified from a pool of multiple HBsAg-positive donor plasma specimens by sucrose gradient centrifugation^14^ and diluted in HBsAg-negative normal human plasma.

#### Human specimens

*Cohort A (n=6)*: acute hepatitis B: seroconversion panels were purchased from North American Biologics Inc. (NABI). These are unique serial samples from 6 patients with very early onset of acute hepatitis B.

*Cohort B (n=113)*: CHB: single time-point plasma specimens were collected from antiviral treatment-naïve patients in Spain and Cameroon through collaborative research studies approved by ethics review boards in each respective country. Specimens were categorized by phases of HBV infection with criteria reported previously.^6^,^15^,^16^ Immune control (IC) phase specimens: HBeAg(−), HBV DNA <2000 IU/mL, qHBsAg <1000 IU/mL or <650 S/CO. CHB specimens: both HBeAg(+) and HBeAg(−) and had HBV DNA >2000 IU/mL. The studies conformed to the ethical guidelines of the 1975 Declaration of Helsinki with approval from the appropriate local institutional review committees.

*Cohort C (n=19)*: CHB on NA therapy: serial serum specimens were collected from HBeAg(+) and HBeAg(−) patients receiving prolonged tenofovir or entecavir in the US. The clinical samples were from a biorepository approved by the Institutional Review Board (IRB) at Beth Israel Deaconess Medical Center (BIDMC).

### Statistics

Group comparisons were calculated using a 2-tailed Student *t* test or ANOVA using Microsoft Office Excel or GraphPad Prism (8.0.2). Significant *p*-value was <0.05.

## RESULTS

Isoform-specific detection was confirmed in prototype assays with recombinant S-HBsAg and Dane particles purified from a multidonor pool that may not reflect actual abundances in a given patient (Table 1). While all 3 assays (T, L, and M) could detect Dane particles; the L-HBsAg and M-HBsAg assays did not detect recombinant S-HBsAg, as expected. HBsAg isoform standards were not available to make the assays quantitative. We, therefore, reported the results as S/N ratios as described in the Methods and human specimens section. HBsAg isoform assay linearity was tested using 2 commercially available HBsAg-positive samples as described in the Methods and human specimens section. Total, M, and L assays showed *R* ^2^ values of 0.998, 1.000, and 0.943 for patient sample 1 and 0.998, 0.999, and 0.840 for patient sample 2, respectively (Supplemental Figure 1, http://links.lww.com/HC9/A83).

**TABLE 1:** Recombinant S-HBsAg and Dane particles purified from a multidonor pool

	L-HBsAg			M-HBsAg			Total HBsAg		
	RLU	S/N	% total	RLU	S/N	% total	RLU	S/N	IU/mL
Recombinant S-HBsAg	53	0.96	NA	50	0.96	NA	366,859	6551	53
Purified Dane virions	733	13.33	0.15	183	3.52	0.04	488,113	8716	81
Normal human plasma	55	1	NA	52	1	NA	56	1	0

Abbreviations: L-HBsAg, large HBsAg; M-HBsAg, middle HBsAg; NA, not applicable; RLU, relative light units; S/N, signal-to-noise.

### Compositions of HBsAg isoforms during acute hepatitis B

**FIGURE 1:**
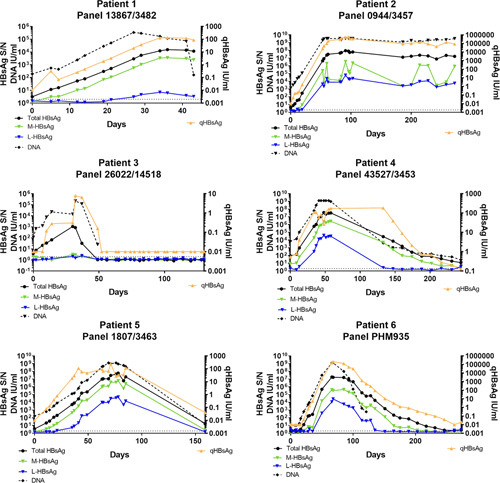
Serial samples from 6 patients with acute hepatitis B from the earliest onset of infection. All cases except patient 2 had spontaneous resolution of the infection with HBsAg clearance. Patient 2 developed chronic hepatitis B with persistently detectable HBsAg beyond 6 months.

To evaluate the changes in individual HBsAg isoform abundances during acute HBV infection, HBsAg isoform, qHBsAg together with HBV DNA levels from serial specimens of 6 patients were measured (Figure 1). All cases, with the exception of patient 2, achieved spontaneous HBsAg clearance. Patient 2 developed CHB with persistent HBsAg beyond 6 months. In all patients, HBsAg was detectable within days of acute infection. The kinetics of T-HBsAg (S/N) and qHBsAg (IU/mL) were similar during the entire course from acute infection to recovery or chronicity. In all the cases, the T-HBsAg, L-HBsAg, and M-HBsAg levels increased in parallel during the initial phase of acute hepatitis B. From the early onset, the S/N of the T-HBsAg was much higher than M-HBsAg and L-HBsAg implying a predominant S-HBsAg isoform. In addition, the change over time of L-HBsAg relative to qHBsAg/T-HBsAg was much smaller in both patients 1 and 3. Patient 3 had very low levels of both M-HBsAg and L-HBsAg throughout the acute and recovery phases. Interestingly, both patients 1 and 3 achieved a clearance of HBsAg, both T-HBsAg and qHBsAg, much earlier compared with the other cases. During resolution of the acute HBV infection, the HBV DNA declined at earlier time points compared with HBsAg.

### Compositions of HBsAg isoforms during CHB

We compared the levels of the HBsAg isoform from single time-point specimens of 113 patients with different phases of CHB (Figure 2A). Among the participants, 41 (36%) were in the IC phase, 36 (32%) had HBeAg(+) CHB, and 36 (32%) had HBeAg(−) CHB. As by definition, the HBV DNA levels were lower among the IC [2.3 log_10_ IU/mL] compared with HBeAg(+) CHB [8.5 log_10_ IU/mL] or HBeAg(−) CHB [5.5 log_10_ IU/mL]. IC had significantly lower qHBsAg titers [2.2 log IU/mL] compared with HBeAg(+) [4.2 log IU/mL] and HBeAg(−) CHB [3.7 log IU/mL]. In each phase of HBV infection, there were considerably wide distributions of HBsAg isoform levels. The average S/N was lowest among IC for all HBsAg isoforms. HBeAg(+) CHB had higher levels of T-HBsAg, L-HBsAg, and M-HBsAg compared with HBeAg(−) CHB. For both IC and CHB patients, the mean M-HBsAg S/N was higher than L-HBsAg.

FIGURE 2(A) Comparison of total qHBsAg (T-HBsAg), qHBsAg, L-HBsAg, M-HBsAg, and HBV DNA titers from single time-point specimens of 113 patients with IC, HBeAg(+) CHB, and HBeAg(−) CHB. Mean values are indicated above each category and with a horizontal line in the plots. *p* Values for comparison of HBeAg(+) CHB versus HBeAg (−) CHB are shown at the top of the plots. *p* Values for comparisons with IC are shown below the plots. (B) The relationship of L-HBsAg and M-HBsAg to T-HBsAg, qHBsAg and HBV DNA for HBeAg(+) and HBeAg(−) CHB patients. Correlations (*R* ^2^) are indicated for HBeAg(+) and (−) CHB patients. Abbreviations: CHB, chronic hepatitis B; IC, immune control; L-HBsAg, large HBsAg; M-HBsAg, middle HBsAg; qHBsAg, quantitative HBsAg.

**Figure:**
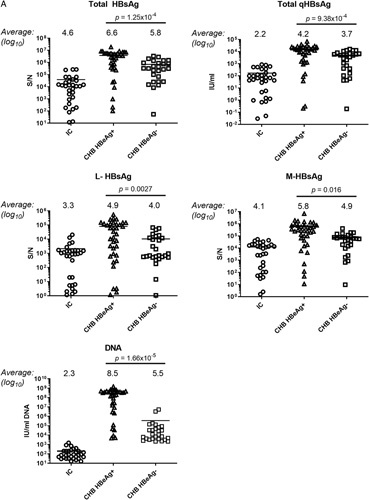

**Figure:**
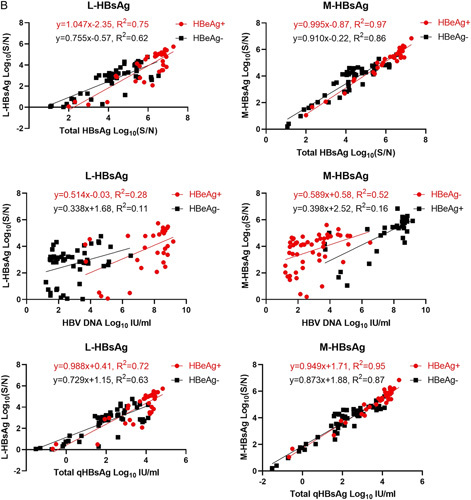

The relationship of M-HBsAg and L-HBsAg to the T-HBsAg, total qHBsAg, and HBV DNA were further evaluated for HBeAg(+) and HBeAg(−) single time-point samples (Figure 2B). While the HBeAg(+) samples had higher S/N for all HBsAg isoforms, the correlations of M-HBsAg and L-HBsAg to either total HBsAg or qHBsAg were similar in HBeAg(+) and HBeAg(−) patients. In contrast, there was no significant correlation between L-HBsAg or M-HBsAg with HBV DNA levels for both HBeAg(+) and HBeAg(−) groups.

### Compositions of HBsAg isoforms during NA therapy

To evaluate the impact of prolonged NA therapy on HBsAg isoform compositions, serial samples from 19 patients on NA were evaluated. The baseline clinical characteristics of these patients are summarized in Table 2. There were 9 patients with HBeAg(+) CHB who achieved HBeAg clearance on therapy and 10 HBeAg(−) CHB patients. HBeAg (+) patients with HBeAg clearance after prolonged therapy were chosen to better evaluate to changes in the HBsAg isoform ratio. The patients were predominantly Asians and were treated with either entecavir or tenofovir for more than 50 months continuously. HBeAg(−) patients were older (36 vs. 48 y, *p* = 0.04). Before therapy, HBeAg(+) patients had significantly higher HBV DNA, HBV RNA, ALT, qHBsAg, T-HBsAg, and M-HBsAg levels. The L-HBsAg levels were similar in the 2 groups. For both groups, the S/N ratio of M-HBsAg was higher than L-HBsAg at baseline.

**TABLE 2:** Baseline clinical features

Baseline patient characteristics	HBeAg loss (n=9)	HBeAg negative (n=10)	*p*
Race	78% Asian, 22% White	100% Asian	NS
Age (y)	36 (23–64)	48 (34–76)	0.04
Sex (female)	66%	30%	0.1
Duration of therapy (mo)	53 (34–67)	52 (19–84)	0.4
HBV DNA (log IU/mL)	7.4 (6.1–8.2)	4.9 (3.4–7.3)	0.0001
HBV RNA (log U/L)	5.8 (1.7–8)	3.2 (1.7–5.7)	0.0014
ALT (U/L)	172 (34–665)	44 (27–70)	0.03
Quantitative HBsAg (log IU/mL)	4.1 (3.1–5.2)	2.7 (2–3.3)	0.0001
L-HBsAg (log S/N)	3.6 (1.3–5.4)	3.1 (1–4.8)	0.1
M-HBsAg (log S/N)	5.4 (3.8–6.7)	3.8 (2.6–4.6)	0.0005
T-HBsAg (log S/N)	6.1 (4.8–7.3)	4.8 (3.8–5.5)	0.0007

Abbreviations: L-HBsAg, large HBsAg; M-HBsAg, middle HBsAg; NS, not significant; S/N, signal-to-noise; T-HBsAg, total HBsAg.

**FIGURE 3:**
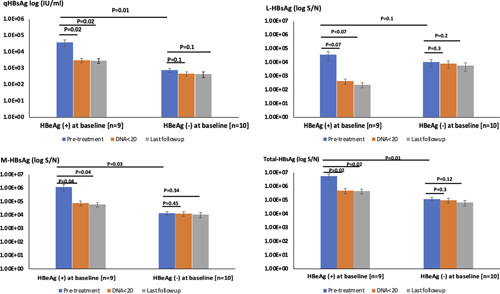
The kinetics of qHBsAg, L-HBsAg, M-HBsAg, and total HBsAg during nucleaos(t)ide analog therapy for HBeAg(+) and HBeAg(−) chronic hepatitis B patients. Comparisons are shown between baseline, at DNA suppression to <20 IU/mL and at last follow-up. Abbreviations: L-HBsAg, large HBsAg; M-HBsAg, middle HBsAg; qHBsAg, quantitative HBsAg.

During therapy, the kinetics of qHBsAg, T-HBsAg, M-HBsAg, and L-HBsAg were similar (Figure 3). For the HBeAg (+) patients, there were significant reductions of the qHBsAg, T-HBsAg, and M-HBsAg by at least 1 log between baseline and time of optimal HBV DNA suppression (*p* <0.05). The mean L-HBsAg also decreased by >1 log but it was only approaching significance (*p* = 0.07). There was no further reduction of the qHBsAg and HBsAg isoforms with prolonged therapy of up to 67 months after HBeAg clearance. For the HBeAg (−) patients, there was no significant reduction in qHBsAg and in any of the HBsAg isoforms from baseline to after achieving HBV DNA <20 IU/mL or at last follow-up. After viral suppression, the L-HBsAg levels appeared lower among HBeAg(+) subjects, but they were not statistically significant.

**FIGURE 4:**
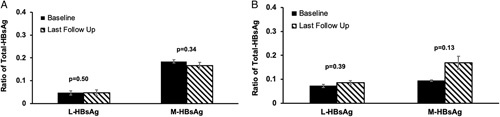
(A) Proportion of L-HBsAg and M-HBsAg to total HBsAg at baseline and at last follow-up of prolonged therapy for patients (n=11) with last quantitative HBsAg >100 IU/mL. (B) Proportion of L-HBsAg and M-HBsAg to total HBsAg at baseline and last follow-up of prolonged therapy for patients (n=7) with last quantitative HBsAg <100 IU/mL. The single patient with HBsAg loss was not included. Abbreviations: L-HBsAg, large HBsAg; M-HBsAg, middle HBsAg.

Eight patients (42%) achieved HBsAg <100 IU/mL at their last follow-up; among them, 1 had HBsAg loss. Three of these patients were HBeAg(+) and 5 were HBeAg(−) before therapy. The patient who had HBsAg loss was HBeAg(+) at baseline. The proportions of L-HBsAg and M-HBsAg to T-HBsAg at baseline and at last follow-up were compared (Figure 4). Among the 11 patients [6 eAg(+), 5 eAg(−) before therapy] who remained HBsAg >100 IU/mL throughout the entire duration of therapy, the proportion of M-HBsAg and L-HBsAg to T-HBsAg were similar between pretreatment and at last follow-up with *p* values of 0.5 and 0.3, respectively (Figure 4A). For those who achieved HBsAg <100 IU/mL, the proportions of L-HBsAg and M-HBsAg were also similar before therapy. At the last follow-up, while the L-HBsAg to T-HBsAg ratio was 0.09, the M-HBsAg to T-HBsAg ratio was 0.17. This last follow-up M-HBsAg ratio was higher compared with baseline in 5 of the 7 subjects, but the mean value did not reach statistical significance (*p* = 0.13) (the one with HBsAg loss was excluded in the analysis) (Figure 4B). The correlation of qHBsAg and M-HBsAg at baseline (*R* ^2^=0.99) was higher than at last follow-up (*R* ^2^=0.68, *p* = 0.089).

**FIGURE 5:**
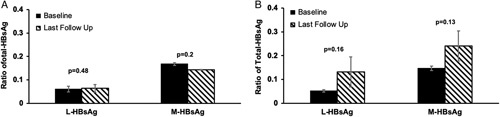
(A) Proportion of L-HBsAg and M-HBsAg to total HBsAg at baseline and at last follow-up of prolonged therapy for patients (n=8) with last HBV RNA >1.65 log U/L. (B) Proportion of L-HBsAg and M-HBsAg to total HBsAg at baseline and at last follow-up of prolonged therapy for patients (n=11) with last HBV RNA <1.65 log U/L. Abbreviations: L-HBsAg, large HBsAg; M-HBsAg, middle HBsAg.

We did a similar analysis to examine the proportions of M-HBsAg and L-HBsAg for those who had HBV RNA <1.65 log U/L (n=11) and ≥1.65 log U/L (n=8) on prolonged therapy (Figure 5). The isoform values were more heterogeneous at last follow-up. The overall trends were similar to the analysis with HBsAg cutoff at 100 IU/mL. The L-HBsAg to T-HBsAg ratios were generally low. The proportions of L-HBsAg were similar at baseline and last follow-up regardless of the final HBV RNA values. The M-HBsAg ratio was higher in 8 of 11 subjects with RNA <1.65 log U/L at last follow-up, but the mean value was not statistically significant (*p* = 0.13)

## DISCUSSION

Total elimination of HBV may not be attainable because the viral covalently closed circular DNA (cccDNA) persists even after spontaneous recovery from acute HBV infection.^17^,^18^ Sustained undetectable HBsAg and HBV DNA in serum, or functional cure, is currently the most feasible goal of hepatitis B management.^17^,^19^ HBsAg circulates in both the virions and the noninfectious SVPs.^20^ The ratio of SVPs to virions is found to be 10,000 to 100,000-fold, while the ratio of SVP filaments to virions is 10-fold.^21^ HBV DNA is known to integrate into the host genome and generates nonreplicative HBV DNA sequences and HBV proteins.^22^ S-HBsAg lacks the ribosomal binding site of L-HBsAg and could be generated by HBV DNA genomes that have integrated at positions downstream of the L-HBsAg start codon.^22^–^24^ Both L-HBsAg and S-HBsAg play essential roles in viral replication. The preS1 region of L-HBsAg, for example, is demonstrated to be critical for viral entry at the sodium-dependent taurocholate cotransporting polypeptide receptor.^4^,^25^ The exact role of the highly conserved M-HBsAg remains poorly understood.

It is noteworthy that the relative levels of L-HBsAg and M-HBsAg to T-HBsAg (both <1% S/N) in our pooled purified Dane particles (Table 1) did not match the proportions of 10% L-HBsAg and 30% M-HBsAg noted in a prep from a single donor on a silver stained gel.^4^ The Third World Health Organization (WHO) international HBsAg standard was also purified from a pool of clinical specimens. Similar to our observations, the WHO standard contains only trace amounts of L-HBsAg and M-HBsAg.^26^,^27^ For the development of these HBsAg isoform assays, we took the approach of measuring signal for each marker separately as each isoform-specific detection antibody may have different sensitivities and is a qualitative result. Therefore, the absolute levels of one isoform may not truly correlate 1:1 to the levels of another isoform in S/N units of each individual assay. Furthermore, the preS1/S2 region of the HBV genome is highly variable^28^ and may prevent isoform-specific antibodies from reliably detecting all genotypes with the same sensitivity. The normalization of the isoform signals to T-HBsAg signal, therefore, is not a reliable measure. We concluded that the S/N ratio was the most reliable unit for comparison of isoform level. The T-HBsAg represents the predominant S-HBsAg.

To our knowledge, the kinetics of the initial rise and subsequent decline of the various HBsAg isoforms during acute HBV infection have not been reported previously. The serial results on the rise of serum qHBsAg and T-HBsAg during the initial 40–50 days of acute infection almost overlapped in all 6 patients (Figure 1). The subsequent decline of qHBsAg and T-HBsAg to baseline within 180 days of acute infection in the 5 patients with spontaneous recovery, and the persistent elevation of qHBsAg and T-HBsAg beyond 200 days in patient 2 with CHB also ran an identical course. These results validated our new HBsAg isoform assays against the well-established quantitative ARCHITECT HBsAg (qHBsAg) assay.

All 3 HBsAg isoforms emerged in parallel within days of acute infection and peaked between 40 and 80 days in all 5 patients with recovery. The serial M-HBsAg levels were persistently higher than L-HBsAg during the early and recovery course but declined to baseline undetectable level at the same time points. This pattern was not well delineated in patient 3 who had very low detectable L-HBsAg and M-HBsAg levels even at the peak of T-HBsAg. In their cross-sectional observations of acute hepatitis B, Pfefferkorn et al.^6^ also noted the presence of a higher proportion of M-HBsAg compared with L-HBsAg. It is hypothesized that M-HBsAg may have a role in modulating immune responses, especially during acute infection, though the mechanism has not been well defined.^29^

A cross-sectional study suggested that the concentrations of the HBsAg isoforms can distinguish between active CHB and inactive HBV infection.^6^ We compared the HBsAg profiles between patients with HBeAg(+) CHB, HBeAg(−) CHB, and those in an IC phase. There was wide-ranging variability of L-HBsAg and M-HBsAg levels in these single time-point specimens (Figure 2). Overall, the results of the qHBsAg and T-HBsAg were in constant agreement. The average levels of T-HBsAg, L-HBsAg, and M-HBsAg were significantly higher among those with HBeAg(+) CHB compared with HBeAg(−) CHB and IC. In addition, the correlations between L-HBsAg and M-HBsAg to T-HBsAg or qHBsAg levels were similar between HBeAg(+) and HBeAg(−) samples. In contrast, there were poor correlations between L-HBsAg and M-HBsAg to HBV DNA levels for either HBeAg(+) or HBeAg(−) cases. In this study, we could not identify absolute cutoff values of L-HBsAg or M-HBsAg that could distinguish between HBeAg(−) CHB and IC due to the highly variable range of single-point levels in each group.

NAs are potent antiviral agents that inhibit the HBV polymerase leading to significant reduction of HBV DNA levels.^16^,^30^ They do not have a direct effect in suppressing cccDNA or integrated HBV DNA.^3^,^16^,^30^ We compared the patterns of HBsAg isoforms on therapy between HBeAg(+) CHB patients who achieved HBeAg loss to HBeAg(−) CHB patients. We described previously the biphasic decline of HBsAg before and after HBeAg clearance in the natural history of HBV infection.^31^ The faster HBsAg reduction before HBeAg loss did not result in shorter duration to HBsAg clearance. After HBeAg seroclearance, the rate of HBsAg decline was similar to that of the HBeAg(−) patients. We hypothesized that the HBeAg(−) patients have a higher proportion of HBsAg generated from the integrated HBV DNA, which may account for the different kinetics of HBsAg decline between HBeAg(+) and HBeAg (−) cases. As expected, patients with HBeAg(+) CHB had significantly higher baseline qHBsAg before therapy. While the pretreatment T-HBsAg and M-HBsAg had a similar trend as the qHBsAg, the L-HBsAg levels were similar between the HBeAg(+) and (−) patients. Among the HBeAg(+) patients, there was a significant reduction in qHBsAg, T-HBsAg, and M-HBsAg from baseline when the HBV DNA was optimally suppressed to <20 IU/mL. Thereafter, there was no significant further decline at last follow-up. In contrast, patients with HBeAg(−) CHB had no significant reduction in all HBsAg isoforms from baseline to last follow-up after similar duration of prolonged therapy >50 months. This biphasic treatment-related HBsAg kinetics with HBeAg(+) CHB was consistent with our prior observations in natural history.

qHBsAg has been applied to predict treatment response with NA.^17^,^19^ Low qHBsAg level at <100 IU/mL has emerged as a useful cutoff value in predicting subsequent HBsAg loss.^19^ Since HBsAg can be produced from cccDNA and integrated HBV DNA, it is less reliable in reflecting the activity of cccDNA.^18^,^22^,^32^ Another novel serum marker is HBV pregenomic RNA which reflects the replicative rate of intrahepatic cccDNA.^33^,^34^ Given that L-HBsAg and M-HBsAg are more abundant in intact virions compared with SVPs, we initially hypothesized that the proportion of these isoforms would be altered after pronged therapy compared with the baseline. In a recent study, the proportion of M-HBsAg at baseline was the best early predictor of HBsAg loss.^7^ We, therefore, further compare the HBsAg isoform profiles of those who achieved optimal qHBsAg (<100 IU/mL) and HBV pregenomic RNA (<1.65 log U/L) after prolonged NA therapy with those who did not. There were no significant differences in the proportions of L-HBsAg between baseline and last follow-up regardless of whether the optimal qHBsAg and HBV pregenomic RNA suppression was achieved. The proportion of the M-HBsAg was noted to be higher among those with optimal qHBsAg and HBV pregenomic RNA suppression at last follow-up but not at baseline though the trend did not reach statistical significance. It is possible that the proportions of L-HBsAg and M-HBsAg to T-HBsAg are relatively small, and it requires a much larger sample size to detect statistical difference.

Accurate disease staging and prediction of antiviral treatment response for CHB will likely require a panel of biomarkers. Our study exemplifies this reality in several aspects. First, HBV DNA titers do not consistently correlate with HBsAg levels. The T-HBsAg assay performed similar to the well-established qHBsAg assay. Individual L-HBsAg or M-HBsAg measurements did not offer additional benefits in predicting resolution of acute hepatitis B or identifying different phases of chronic HBV infection. The enhanced ratio of M-HBsAg to T-HBsAg with prolonged NA therapy may reflect SVP produced from integrated HBV DNA and deserve further evaluation with a larger sample size. The roles of HBsAg isoform in predicting functional cure, especially with novel direct-acting antiviral therapies, need further evaluation.

## Supplementary Material

## References

[1] Ganem D Prince AM. Hepatitis B virus infection—natural history and clinical consequences. N Engl J Med. 2004;350:1118–29. 15014185 10.1056/NEJMra031087

[2] Wang J Huang H Liu Y Chen R Yan Y Shi S. HBV Genome and Life Cycle. Adv Exp Med Biol. 2020;1179:17–37. 31741332 10.1007/978-981-13-9151-4_2

[3] Yuen MF Chen DS Dusheiko GM Janssen HLA Lau DTY Locarnini SA. Hepatitis B virus infection. Nat Rev Dis Primers. 2018;4:18035. 29877316 10.1038/nrdp.2018.35

[4] Heermann KH Goldmann U Schwartz W Seyffarth T Baumgarten H Gerlich WH. Large surface proteins of hepatitis B virus containing the pre-s sequence. J Virol. 1984;52:396–402. 6492255 10.1128/jvi.52.2.396-402.1984 PMC254539

[5] Gerlich WH. Medical virology of hepatitis B: how it began and where we are now. Virol J. 2013;10:239. 23870415 10.1186/1743-422X-10-239 PMC3729363

[6] Pfefferkorn M Bohm S Schott T Deichsel D Bremer CM Schroder K. Quantification of large and middle proteins of hepatitis B virus surface antigen (HBsAg) as a novel tool for the identification of inactive HBV carriers. Gut. 2018;67:2045–2053. 28951526 10.1136/gutjnl-2017-313811

[7] Pfefferkorn M Schott T Böhm S Deichsel D Felkel C Gerlich WH. Composition of HBsAg is predictive of HBsAg loss during treatment in patients with HBeAg-positive chronic hepatitis B. J Hepatol. 2021;74:283–292. 32931877 10.1016/j.jhep.2020.08.039

[8] Rinker F Bremer CM Schröder K Wiegand SB Bremer B Manns MP. Quantitation of large, middle and small hepatitis B surface proteins in HBeAg-positive patients treated with peginterferon alfa-2a. Liver Int. 2020;40:324–332. 31721419 10.1111/liv.14298

[9] Brancaccio G Salpini R Piermatteo L Surdo M Fini V Colagrossi L. An Increase in the Levels of Middle Surface Antigen Characterizes Patients Developing HBV-Driven Liver Cancer Despite Prolonged Virological Suppression. Microorganisms. 2021;9:752; PMID: 33918474. 33918474 10.3390/microorganisms9040752 PMC8065957

[10] Bazinet M Anderson M Pantea V Placinta G Moscalu I Cebotarescu V. HBsAg isoform dynamics during NAP-based therapy of HBeAg-negative chronic HBV and HBV/HDV infection. Hepatol Commun. 2022;6:1870–1880. 35368148 10.1002/hep4.1951 PMC9315123

[11] Lou S Taylor R Pearce S Kuhns M Leary T. An ultra-sensitive Abbott ARCHITECT((R)) assay for the detection of hepatitis B virus surface antigen (HBsAg). J Clin Virol. 2018;105:18–25. 29843004 10.1016/j.jcv.2018.05.009

[12] Mimms LT Floreani M Tyner J Whitters E Rosenlof R Wray L. Discrimination of hepatitis B virus (HBV) subtypes using monoclonal antibodies to the PreS1 and PreS2 domains of the viral envelope. Virology. 1990;176:604–619. 1693248 10.1016/0042-6822(90)90031-l

[13] Butler EK Gersch J McNamara A Luk KC Holzmayer V de Medina M. Hepatitis B Virus Serum DNA andRNA Levels in Nucleos(t)ide Analog-Treated or Untreated Patients During Chronic and Acute Infection. Hepatology. 2018;68:2106–2117. 29734472 10.1002/hep.30082

[14] Kimura T Rokuhara A Matsumoto A Yagi S Tanaka E Kiyosawa K. New enzyme immunoassay for detection of hepatitis B virus core antigen (HBcAg) and relation between levels of HBcAg and HBV DNA. J Clin Microbiol. 2003;41:1901–1906. 12734224 10.1128/JCM.41.5.1901-1906.2003 PMC154683

[15] Invernizzi F Vigano M Grossi G Lampertico P. The prognosis and management of inactive HBV carriers. Liver Int. 2016;36(Suppl 1):100–104. 10.1111/liv.13006 26725905

[16] EASL 2017 Clinical Practice Guidelines on the management of hepatitis B virus infection. J Hepatol. 2017;67:370–98. 28427875 10.1016/j.jhep.2017.03.021

[17] Cornberg M Lok AS Terrault NA Zoulim F. Guidance for design and endpoints of clinical trials in chronic hepatitis B - Report from the 2019 EASL-AASLD HBV Treatment Endpoints Conference(‡). J Hepatol. 2020;72:539–557. 31730789 10.1016/j.jhep.2019.11.003

[18] Lai CL Wong D Ip P Kopaniszen M Seto WK Fung J. Reduction of covalently closed circular DNA with long-term nucleos(t)ide analogue treatment in chronic hepatitis B. J Hepatol. 2017;66:275–281. 27639844 10.1016/j.jhep.2016.08.022

[19] Liaw YF. Clinical utility of HBV surface antigen quantification in HBV e antigen-negative chronic HBV infection. Nat Rev Gastroenterol Hepatol. 2019;16:631–41. 31477873 10.1038/s41575-019-0197-8

[20] Lesmana CR Jackson K Lim SG Sulaiman A Pakasi LS Gani RA. Clinical significance of hepatitis B virion and SVP productivity: relationships between intrahepatic and serum markers in chronic hepatitis B patients. United European Gastroenterol J. 2014;2:99–107. 10.1177/2050640614525151 PMC4040813 24918014

[21] Chai N Chang HE Nicolas E Han Z Jarnik M Taylor J. Properties of subviral particles of hepatitis B virus. J Virol. 2008;82:7812–7817. 18524834 10.1128/JVI.00561-08 PMC2519590

[22] Wooddell CI Yuen MF Chan HL Gish RG Locarnini SA Chavez D. RNAi-based treatment of chronically infected patients and chimpanzees reveals that integrated hepatitis B virus DNA is a source of HBsAg. Sci Transl Med. 2017;9:eaan0241; PMID: 28954926. 28954926 10.1126/scitranslmed.aan0241 PMC5830187

[23] Svicher V Salpini R Piermatteo L Carioti L Battisti A Colagrossi L. Whole exome HBV DNA integration is independent of the intrahepatic HBV reservoir in HBeAg-negative chronic hepatitis B. Gut. 2021;70:2337–2348. 33402415 10.1136/gutjnl-2020-323300 PMC8588301

[24] Tu T Budzinska MA Shackel NA Urban S. HBV DNA Integration: Molecular Mechanisms and Clinical Implications. Viruses. 2017;9:75; PMID: 28394272. 28394272 10.3390/v9040075 PMC5408681

[25] Pfaff E Klinkert MQ Theilmann L Schaller H. Characterization of large surface proteins of hepatitis B virus by antibodies to preS-S encoded amino acids. Virology. 1986;148:15–22. 3510505 10.1016/0042-6822(86)90399-5

[26] Wilkinson DE Seiz PL Schuttler CG Gerlich WH Glebe D Scheiblauer H. International collaborative study on the 3rd WHO International Standard for hepatitis B surface antigen. J Clin Virol. 2016;82:173–180. 27345251 10.1016/j.jcv.2016.06.003

[27] Seiz PL Mohr C Wilkinson DE Ziebuhr J Schuttler CG Gerlich WH. Characterization of the 3rd International Standard for hepatitis B virus surface antigen (HBsAg). J Clin Virol. 2016;82:166–172. 27345250 10.1016/j.jcv.2016.05.009

[28] Pollicino T Cacciola I Saffioti F Raimondo G. Hepatitis B virus PreS/S gene variants: pathobiology and clinical implications. J Hepatol. 2014;61:408–417. 24801416 10.1016/j.jhep.2014.04.041

[29] Schmitt S Glebe D Tolle TK Lochnit G Linder D Geyer R. Structure of pre-S2 N- and O-linked glycans in surface proteins from different genotypes of hepatitis B virus. J Gen Virol. 2004;85:2045–2053. 15218190 10.1099/vir.0.79932-0

[30] Terrault NA Lok ASF McMahon BJ Chang KM Hwang JP Jonas MM. Update on prevention, diagnosis, and treatment of chronic hepatitis B: AASLD 2018 hepatitis B guidance. Hepatology. 2018;67:1560–1599. 29405329 10.1002/hep.29800 PMC5975958

[31] Shah PA Kaur S Simons B Talat A McMahon B Lau DTY. Patterns of HBeAg and HBsAg clearance among a treatment-naive Alaskan Cohort in a long-term observational study. J Viral Hepat. 2020;27:644–646. 32043714 10.1111/jvh.13271

[32] Gong SS Jensen AD Wang H Rogler CE. Duck hepatitis B virus integrations in LMH chicken hepatoma cells: identification and characterization of new episomally derived integrations. J Virol. 1995;69:8102–8108. 7494330 10.1128/jvi.69.12.8102-8108.1995 PMC189762

[33] Su Q Wang SF Chang TE Breitkreutz R Hennig H Takegoshi K. Circulating hepatitis B virus nucleic acids in chronic infection: representation of differently polyadenylated viral transcripts during progression to nonreplicative stages. Clin Cancer Res. 2001;7:2005–2015. 11448918

[34] van Bömmel F Bartens A Mysickova A Hofmann J Krüger DH Berg T. Serum hepatitis B virus RNA levels as an early predictor of hepatitis B envelope antigen seroconversion during treatment with polymerase inhibitors. Hepatology. 2015;61:66–76. 25132147 10.1002/hep.27381

