# Supplementary figures and images for: Characterization of HBV surface antigen isoforms in the natural history and treatment of HBV infection
Source: Hepatol Commun. 2023 Apr 4;7(4):e0027. doi: 10.1097/HC9.0000000000000027 (PMC10079349; doi:10.1097/HC9.0000000000000027)

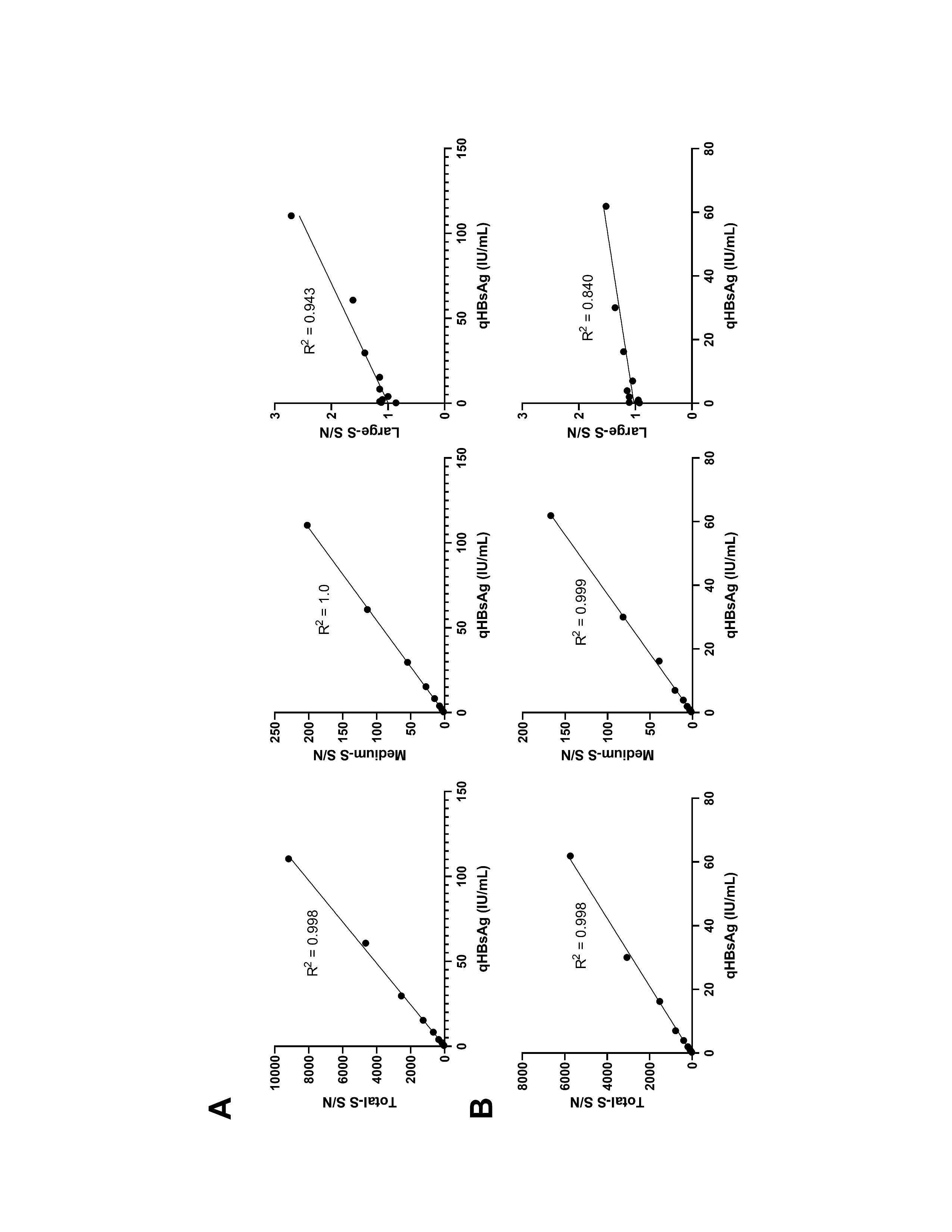

Supplement: Supplementary file 1 [file hc9-7-e0027-s001.tiff]
